# A concise synthesis of (±)-7-*O*-galloyltricetiflavan†

**DOI:** 10.1039/c8ra01606b

**Published:** 2018-04-18

**Authors:** Wenxuan Zhang, Wenjie Xue, Yuqing Jia, Gang Wen, Xu Lian, Jing Shen, Ailin Liu, Song Wu

**Affiliations:** State Key Laboratory of Bioactive Substance and Function of Natural Medicines, Institute of Materia Medica, Chinese Academy of Medical Sciences and Peking Union Medical College Beijing 100050 People's Republic of China ws@imm.ac.cn

## Abstract

(±)-7-*O*-galloyltricetiflavan (1a) was synthesized successfully in five steps from the commercially available trihydroxyacetophenone (2) and trimethoxybenzoyl chloride (3). The flavone 4a was prepared in a one-pot reaction and it gave hex-*O*-methylflavan 6 followed by acylation and reduction. However, the demethylation of flavan 6, 5-*O*-acetylflavan 10 and 5-*O*-phenylacetylflavan 11 by BBr_3_ gave all the hydrolyzed fragments 7 and 8 as the major products. By contrast, in the same condition, hept-*O*-methylflavan 9 could provide the desired product (±)-7-*O*-galloyltricetiflavan (1a) in 91% yield. The additional 5-*O*-B-Br_2_ complex may stabilize the ester bond during the demethylation process.

## Abstract 2

We report the first total synthesis of (±)-7-*O*-galloyltricetiflavan (1a) in five steps as well as an interesting discovery during the demethylation process.

## Graphical abstract

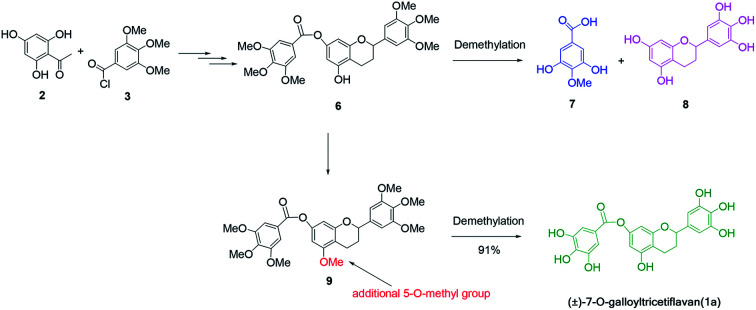

(−)-7-*O*-Galloyltricetiflavan (1, Fig. 1) was isolated from a methanolic extract of the leaves of *Pithecellobium clypearia* by Ooi V. and coworkers in 2006.^1^ It is a catechin-like compound without an OH substituent at C-3, and it shows good antiviral activities against respiratory syncytial virus (RSV), influenza A (H1N1) virus, coxsackie B3 (Cox B3) virus and herpes simplex virus type 1 (HSV-1) as well as anti-inflammatory and anti-allergic activities.^2–5^ To date, the preparation of (−)-7-*O*-galloyltricetiflavan (1) still requires extraction and purification of plant material, and only a few synthetic examples of this type of flavan have been reported.^6,7^ Herein we report the first total synthesis of (±)-7-*O*-galloyltricetiflavan (1a) in five steps as well as an interesting discovery during the demethylation process.

**Fig. 1:**
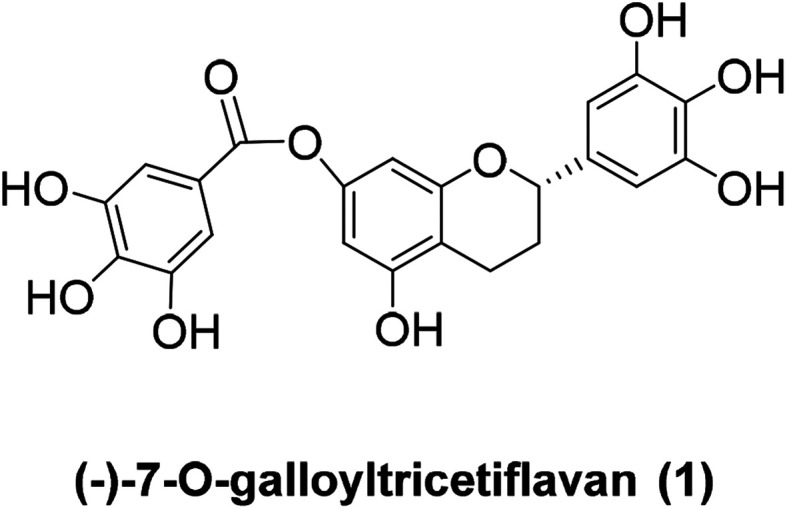
The structure of 7-*O*-galloyltricetiflavan (1).

The synthesis of (±)-7-*O*-galloyltricetiflavan (1a) was started from the preparation of the flavone derivative 4a as shown in Scheme 1. Buckle's group reported an efficient one-pot synthesis of flavones by the treatment of 2-hydroxyacetophenones with the corresponding aroyl chloride in wet K_2_CO_3_/acetone (1% w/w water),^8^ but the reaction proceeded very slowly because the trihydroxyacetophenone (2) was insoluble in acetone. With water-toluene as the solvent, in the presence of K_2_CO_3_ and tetrabutylammonium hydrogen sulfate,^9^ the reaction could provide flavone 4a in 30% yield and 3-acylated product 4b in 50% yield in one-pot in about two hours. Many efforts to improve the yield of 4a failed, but 4b could be converted into 4a by hydrolysis in 50% yield.^10^ Afterwards, acylation of 4a with trimethoxybenzoyl chloride (3) and K_2_CO_3_ gave 7-*O*-galloylflavone 5 in 91% yield, which was then reduced to the flavan 6 by hydrogenation with palladium on carbon as the catalyst for 3 days in 62% yield.^11^

**Scheme 1:**
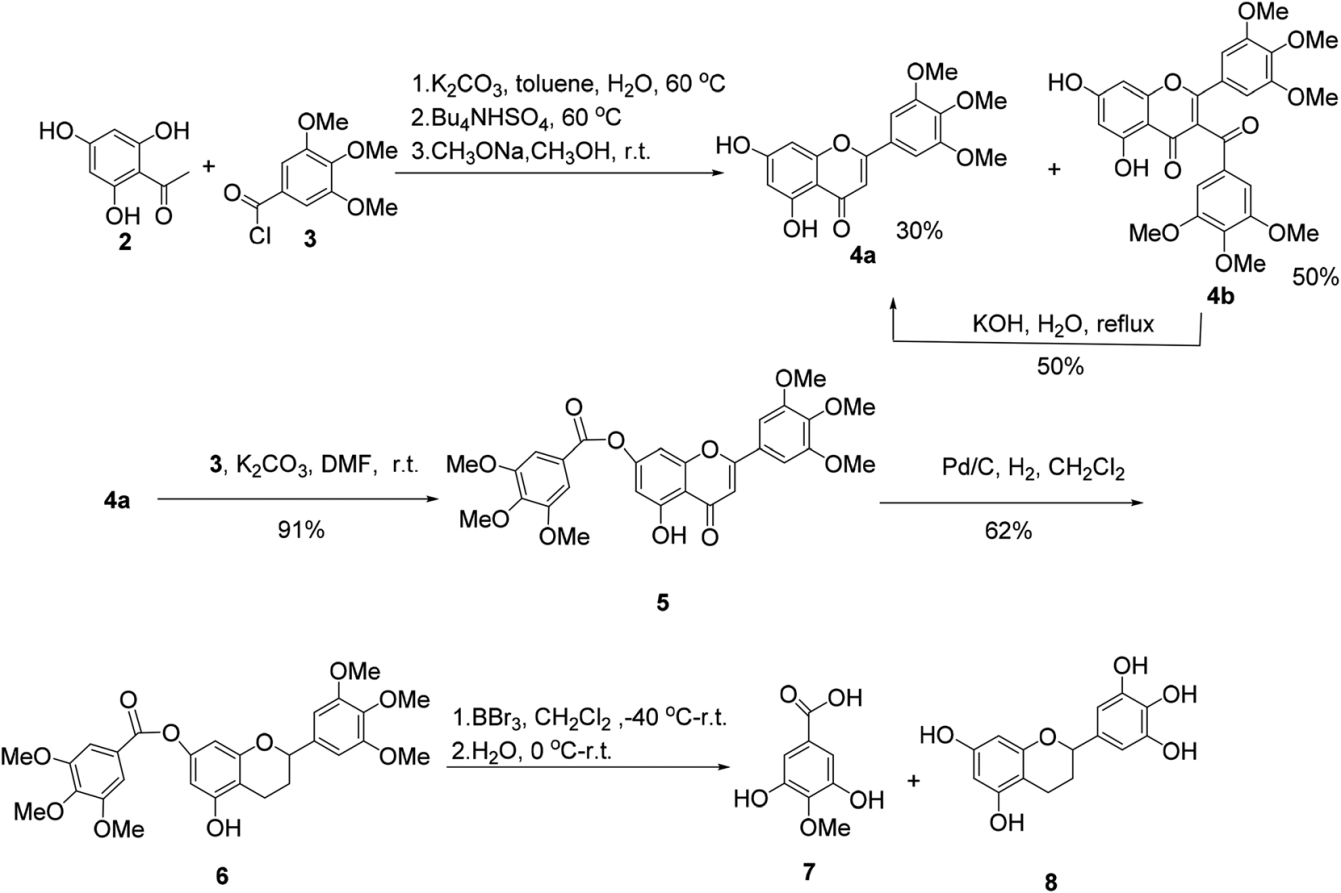
The synthesis of intermediates of (±)-7-*O*-galloyltricetiflavan (1a).

When flavan 6 was treated with BBr_3_ in dichloromethane at −40 °C or −78 °C,^12^ the desired product 1a was generated in only 3% yield (based on HPLC-MS analysis), accompanied with 4-*O*-methyl gallic acid (7) and flavan 8 as the major products, indicating the ester bond of hex-*O*-methylflavan 6 is highly unstable under acidic conditions (Scheme 1).

Then, 5-*O*-methylflavan 9, 5-*O*-acetylflavan 10 and 5-*O*-phenylacetylflavan 11 were prepared as substrates to explore if they provided different results (Scheme 2). Similarly, 5-*O*-acetylflavan 10 and 5-*O*-phenylacetylflavan 11 were not tolerated under these reaction conditions, which gave the hydrolyzed products 7 and 8 as major products. In contrast, when flavan 9 was treated with BBr_3_ in dichloromethane at −40 °C to room temperature, the desired product (±)-7-*O*-galloyltricetiflavan (1a) was generated in 91% yield and no hydrolyzed product was detected after 24 h. The structure of (±)-7-*O*-galloyltricetiflavan (1a) were confirmed by ^1^H NMR, ^13^C NMR, and HR-MS spectrum, and they are consistent with the literature's report.^1^

We presumed that when BBr_3_ was added to the additional 5-*O*-methyl group to form the 5-*O*-B-Br_2_ complex, it may stabilizes the ester bond of 7-phenolic hydroxyl group. By contrast, the 5-*O*-acetyl or 5-*O*-phenylacetyl groups was more easily hydrolyzed and could not help stabilize the ester bond.

In conclusion, (±)-7-*O*-galloyltricetiflavan (1a) was synthesized successfully in five steps from commercial available trihydroxyacetophenone (2) and trimethoxybenzoyl chloride (3). Flavone 4a was prepared in a one-pot reaction and it gave hex-*O*-methylflavan 6 followed by acylation and reduction. However, the demethylation of flavan 6 by BBr_3_ gave the hydrolyzed fragments 7 and 8 as major products. Similarly, neither 5-*O*-acetylflavan 10 nor 5-*O*-phenylacetylflavan 11 could provide the desired product. In contrast, hept-*O*-methylflavan 9 could give the desired product (±)-7-*O*-galloyltricetiflavan (1a) in 91% yield. The additional 5-*O*-B-Br_2_ complex may stabilize the ester bond during the demethylation process. Our method could also provide an efficiently pathway to prepare other 7-*O*-acylated flavans.

**Scheme 2:**
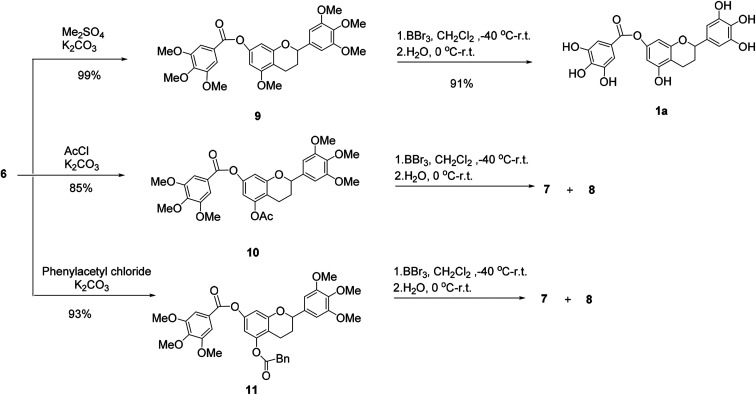
The demethylation of flavan derivatives.

## Experimental section

### General experimental procedures

All reactions were performed in glassware containing a Teflon-coated stir bar. Solvents and chemical reagents were obtained from commercial sources and used without further purification. ^1^H and ^13^C NMR spectra were recorded on Varian Mercury 500 MHz or 400 MHz, and the data were recorded using DMSO-*d*_6_, CDCl_3_ and CD_3_OD as the solvents. Chemical shifts (*δ*) are reported in ppm downfield from an internal TMS standard. The reactions and products were analyzed by HPLC-MS. High-resolution mass spectra were obtained in ESI mode on a hybrid IT-TOF mass spectrometer. Flash column chromatography on silica gel (200–300 mesh) was used for the routine purification of reaction products. The column output was monitored by TLC on silica gel (100–200 mesh) precoated on glass plates (15 × 50 mm), and spots were visualized by a 5% vanillin sulfuric acid/ethanol solution.

#### Synthesis of compounds 4a and 4b

The two-phase mixture of a trihydroxyacetophenone (2, 4.0 g, 21.06 mmol) and an aroyl chloride (3, 13.34 g, 56.14 mmol) in toluene (100 mL) and saturated aqueous K_2_CO_3_ (100 mL) was vigorously stirred at 60 °C for 30 min. Tetrabutylammonium hydrogen sulfate (7.3 g, 21.5 mmol) was added, and the mixture was stirred at 75 °C for an additional two hours. During this period, the organic layer turned orange, and an orange-brown liquid separated at the interface. The toluene layer was separated, and the orange-brown liquid was extracted with CHCl_3_ (50 mL). The toluene and CHCl_3_ solutions were washed with water (2 × 90 mL), dried with Na_2_SO_4_, filtrated and concentrated to give a brown oil (15.2 g). The mixture was redissolved in methanol (60 mL), CH_3_ONa (2.32 g) was added, the solution was then stirred for 30 min at r.t. until 2 M HCl (aq, 18 mL) was added to adjust the pH to 6–7. The resulting yellow solid was isolated by filtration and washed with 40 mL CHCl_3_ to give compound 4a as a canary yellow solid (2.19 g) in 30% yield. The CHCl_3_ solution was concentrated to give compound 4b as a yellow solid (5.76 g) in 50% yield.

#### 5,7-Dihydroxy-2-(3,4,5-trimethoxyphenyl)-4*H*-chromen-4-one (4a)

^1^H NMR (500 MHz, DMSO-*d*_6_) *δ* 12.89 (s, 1H), 10.88 (s, 1H), 7.37 (s, 2H), 7.10 (s, 1H), 6.61 (d, *J* = 2.1 Hz, 1H), 6.25 (d, *J* = 2.1 Hz, 1H), 3.94 (s, 6H), 3.78 (s, 3H). ^13^C NMR (100 MHz, DMSO-*d*_6_) *δ* 182.4, 164.8, 163.5, 161.9, 157.9, 153.7, 141.2, 126.5, 105.5, 104.6, 104.3, 99.4, 94.8, 60.7, 56.8, 56.5, 19.0. HRMS-ESI (*m*/*z*): [M + Na^+^] calcd for C_18_H_16_NaO_7_ 367.0788; found 367.0781.

#### 5,7-Dihydroxy-3-(3,4,5-trimethoxybenzoyl)-2-(3,4,5-trimethoxyphenyl)-4*H*-chromen-4-one (4b)

^1^H NMR (500 MHz, DMSO-*d*_6_) ^1^H NMR (500 MHz, DMSO-*d*_6_) *δ* 12.38 (s, 1H), 11.05 (s, 1H), 7.27 (s, 2H), 6.92 (s, 2H), 6.59 (s, 1H), 6.31 (s, 1H), 3.75 (s, 3H), 3.68 (s, 3H), 3.65 (s, 7H). ^13^C NMR (125 MHz, DMSO-*d*_6_) *δ* 191.7, 180.5, 165.5, 162.7, 162.1, 158.1, 153.7, 153.4, 143.4, 140.5, 132.8, 127.1, 120.5, 107.5, 106.8, 104.1, 99.9, 95.2, 60.9, 60.8, 57.0, 56.4. HRMS-ESI (*m*/*z*): [M + Na^+^] calcd for C_28_H_26_NaO_11_ 561.1367; found 561.1369.

#### 5-Hydroxy-4-oxo-2-(3,4,5-trimethoxyphenyl)-4*H*-chromen-7-yl-3,4,5-trimethoxy-benzoate (5)

To a solution of compound 4a (2.15 g, 6.24 mmol) in 50 mL of dry DMF was added aroyl chloride 3 (4.32 g, 18.73 mmol) and anhydrous K_2_CO_3_ (1.73 g, 12.51 mmol), and the mixture was stirred for 30 min and adjusted to pH = 5 with 2 M HCl (aq). The solid was precipitated, filtrated, washed with DMF and water and then dried at 50 °C to give compound 5 (3.03 g) as a brown solid in 91% yield.

^1^H NMR (500 MHz, CDCl_3_) *δ* 12.78 (s, 1H), 7.44 (s, 2H), 7.10 (s, 2H), 7.01 (s, 1H), 6.70 (d, *J* = 3.7 Hz, 2H), 3.95 (d, *J* = 6.6 Hz, 18H). ^13^C NMR (101 MHz, CDCl_3_) *δ* 182.8, 164.6, 164.0, 162.0, 156.7, 156.3, 153.7, 153.2, 141.7, 126.1, 123.5, 108.9, 107.6, 105.9, 105.7, 103.8, 101.3, 61.1, 61.1, 56.4. HRMS-ESI (*m*/*z*): [M + Na^+^] calcd for C_28_H_26_NaO_11_ 561.1367; found 561.1373.

#### 5-Hydroxy-2-(3,4,5-trimethoxyphenyl)chroman-7-yl-3,4,5-trimethoxybenzoate (6)

To a solution of compound 5 (2.03 g, 3.77 mmol) in 150 mL of CH_2_Cl_2_ was added palladium on carbon (5%, 1.00 g), and the mixture was stirred for 72 h under H_2_ at 0.40 MPa pressure at r.t. The solution was filtrated, concentrated, and then purified by silica gel column chromatography to give compound 6 (1.23 g) as a white foam solid in 62% yield.

^1^H NMR (500 MHz, CDCl_3_) *δ* 7.47 (s, 2H), 6.69 (s, 2H), 6.45 (s, 1H), 6.31 (s, 1H), 4.98 (d, *J* = 10.5 Hz, 1H), 3.97 (s, 6H), 3.92 (s, 6H), 3.90 (s, 3H), 2.87 (d, *J* = 16.0 Hz, 1H), 2.82–2.70 (m, 2H), 2.29 (d, *J* = 13.1 Hz, 1H), 2.15–2.03 (m, 2H). ^13^C NMR (100 MHz, CDCl_3_) *δ* 165.2, 156.6, 154.7, 153.4, 153.1, 149.8, 142.9, 137.6, 137.0, 124.3, 107.5, 103.1, 102.8, 101.3, 78.0, 61.0, 60.9, 56.4, 56.2, 29.5, 19.6. HRMS-ESI (*m*/*z*): [M + Na^+^] calcd for C_28_H_30_NaO_10_ 549.1731; found 549.1758.

#### 5-Methoxy-2-(3,4,5-trimethoxyphenyl)chroman-7-yl-3,4,5-trimethoxybenzoate (9)

To a solution of compound 6 (600 mg, 1.14 mmol) in 5 mL of dry acetone was added dimethyl sulfate (238 μL, 2.51 mmol) and anhydrous K_2_CO_3_ (472 mg, 3.42 mmol), and the mixture was stirred for 5 h at 56 °C. The solution was concentrated, redissolved in EtOAc washed with water, dried with Na_2_SO_4_, filtered, and then concentrated to give a brown oil. After purification by flash silica gel column chromatography, compound 9 (0.62 g) was obtained as a white solid in 99.0% yield.

^1^H NMR (400 MHz, CDCl_3_) *δ* 7.48 (s, 2H), 6.70 (s, 2H), 6.49 (d, *J* = 2.2 Hz, 1H), 6.36 (d, *J* = 2.2 Hz, 1H), 4.98 (dd, *J* = 10.8, 2.1 Hz, 1H), 3.98 (d, *J* = 1.0 Hz, 9H), 3.92 (s, 6H), 3.89 (s, 3H), 3.87 (s, 3H), 2.97–2.82 (m, 1H), 2.82–2.67 (m, 2H), 2.34–2.21 (m, 1H), 2.08–1.99 (m, 2H).^13^C NMR (100 MHz, CDCl_3_) *δ* 165.0, 158.4, 156.1, 153.4, 153.1, 150.2, 142.8, 137.6, 137.2, 137.2, 124.5, 108.7, 107.4, 103.2, 103.1, 96.9, 78.0, 61.0, 60.9, 60.4, 56.4, 56.2, 55.7, 29.7, 29.6, 19.8, 14.2. HRMS-ESI (*m*/*z*): [M + Na^+^] calcd for C_29_H_32_NaO_11_ 563.1888; found 563.1892.

#### Synthesis of (±)-7-*O*-galloyltricetiflavan (1a)

Compound 9 (0.48 g, 0.88 mmol) was dissolved in dry CH_2_Cl_2_ (20 mL), then BBr_3_ (15.4 mL, 1.0 M in CH_2_Cl_2_) was added dropwise at −40 °C. The resultant red-brown solution was warmed to r.t. and stirred for 12 h under N_2_. Upon completion, the reaction contents were quenched by the addition of ice water (20 mL), the CH_2_Cl_2_ was removed, and the water layer was extracted twice with ethyl acetate (50 mL). The combined organic extracts were then washed with water (25 mL) and brine (15 mL), dried with Na_2_SO_4_, filtered, and concentrated. The residue was purified over Sephadex LH-20 gel to give (±)-7-*O*-galloyltricetiflavan (0.36 g) as a brown solid in 91% yield and in 98% purity (from HPLC-MS).

^1^H NMR (500 MHz, CD_3_OD) *δ* 7.21 (s, 2H), 6.47 (s, 2H), 6.22 (d, *J* = 11.9 Hz, 2H), 4.84 (s, 1H), 2.79 (d, *J* = 17.2 Hz, 1H), 2.74–2.65 (m, 1H), 2.27–2.15 (m, 1H), 2.09–1.95 (m, 1H). ^13^C NMR (100 MHz, CD_3_OD) *δ* 167.0, 157.7, 157.2, 151.4, 146.8, 146.5, 140.3, 133.9, 133.6, 120.7, 110.4, 108.5, 106.1, 102.3, 101.4, 79.0, 30.4, 20.3. HRMS-ESI (*m*/*z*): [M − H^−^] calcd for C_22_H_19_O_10_ 441.0827; found 441.0825.

#### 5-Methoxy-2-(3,4,5-trimethoxyphenyl)chroman-7-yl-3,4,5-trimethoxybenzoate (10)

To a solution of compound 6 (100 mg, 0.19 mmol) in 5 mL of dry acetone was added acetyl chloride (25 μL) and anhydrous K_2_CO_3_ (52 mg, 0.38 mmol), and the mixture was stirred for 2 h and adjusted to pH = 5 with 2 M HCl (aq). The solution was concentrated, redissolved in EtOAc washed with water, dried with Na_2_SO_4_, filtered and concentrated to give a brown oil. After purification by silica gel column chromatography, compound 10 (91 mg) was obtained as a colorless oil in 85% yield.

^1^H NMR (500 MHz, CDCl_3_) *δ* 7.42 (s, 2H), 6.74 (d, *J* = 2.2 Hz, 1H), 6.64 (s, 2H), 6.61 (d, *J* = 2.3 Hz, 1H), 4.98 (d, *J* = 10.6 Hz, 1H), 3.99–3.80 (m, 18H), 2.78–2.67 (m, 2H), 2.22 (d, *J* = 14.0 Hz, 1H), 2.06 (dt, *J* = 23.4, 9.5 Hz, 1H). ^13^C NMR (100 MHz, CDCl_3_) *δ* 168.8, 164.5, 156.4, 153.4, 153.1, 149.7, 149.5, 142.9, 137.7, 136.7, 124.2, 113.0, 108.5, 108.1, 107.4, 103.1, 78.2, 77.3, 61.0, 60.9, 56.4, 56.2, 56.2, 29.2, 20.8, 20.1. HRMS-ESI (*m*/*z*): [M + Na^+^] calcd for C_30_H_32_NaO_11_ 591.1837; found 591.1849.

#### 5-(2-Phenylacetoxy)-2-(3,4,5-trimethoxyphenyl)chroman-7-yl-3,4,5-trimethoxy-benzoate (11)

To a solution of compound 6 (100 mg, 0.19 mmol) in 5 mL of dry acetone of was added phenylacetyl chloride (50 μL) and anhydrous K_2_CO_3_ (52 mg, 0.38 mmol), and the mixture was stirred for 2 h and adjusted to pH = 5 with 2 M HCl (aq). The solution was concentrated, redissolved in EtOAc washed with water, dried with Na_2_SO_4_, filtered, and concentrated to give a brown oil. After purification by silica gel column chromatography, compound 11 (110 mg) was obtained as a colorless oil in 93% yield.

^1^H NMR (500 MHz, CDCl_3_) *δ* 7.54–7.33 (m, 7H), 6.76 (d, *J* = 2.3 Hz, 1H), 6.66 (s, 2H), 6.62 (d, *J* = 2.1 Hz, 1H), 4.97 (d, *J* = 10.3 Hz, 1H), 4.04–3.86 (m, 18H), 2.59 (dd, *J* = 10.7, 5.8 Hz, 2H), 2.23–2.11 (m, 1H), 2.09–1.94 (m, 2H). ^13^C NMR (100 MHz, CDCl_3_) *δ* 169.3, 164.6, 156.4, 153.4, 153.1, 149.6, 149.4, 142.9, 137.7, 136.6, 133.3, 129.4, 128.8, 128.8, 127.5, 124.2, 113.0, 108.5, 108.1, 107.4, 103.1, 78.2, 77.3, 61.0, 60.9, 56.4, 56.2, 41.4, 29.2, 19.9. HRMS-ESI (*m*/*z*): [M + Na^+^] calcd for C_36_H_36_NaO_11_ 667.2150; found 667.2165.

## Conflicts of interest

There are no conflicts to declare.

## Supplementary Material

RA-008-C8RA01606B-s001
