# Supplementary material for: A concise synthesis of (±)-7-O-galloyltricetiflavan
Source: RSC Adv. 2018 Apr 18;8(26):14389–92. doi: 10.1039/c8ra01606b (PMC9079901; doi:10.1039/c8ra01606b)

Electronic Supplementary Material (ESI) for RSC Advances.
This journal is © The Royal Society of Chemistry 2018

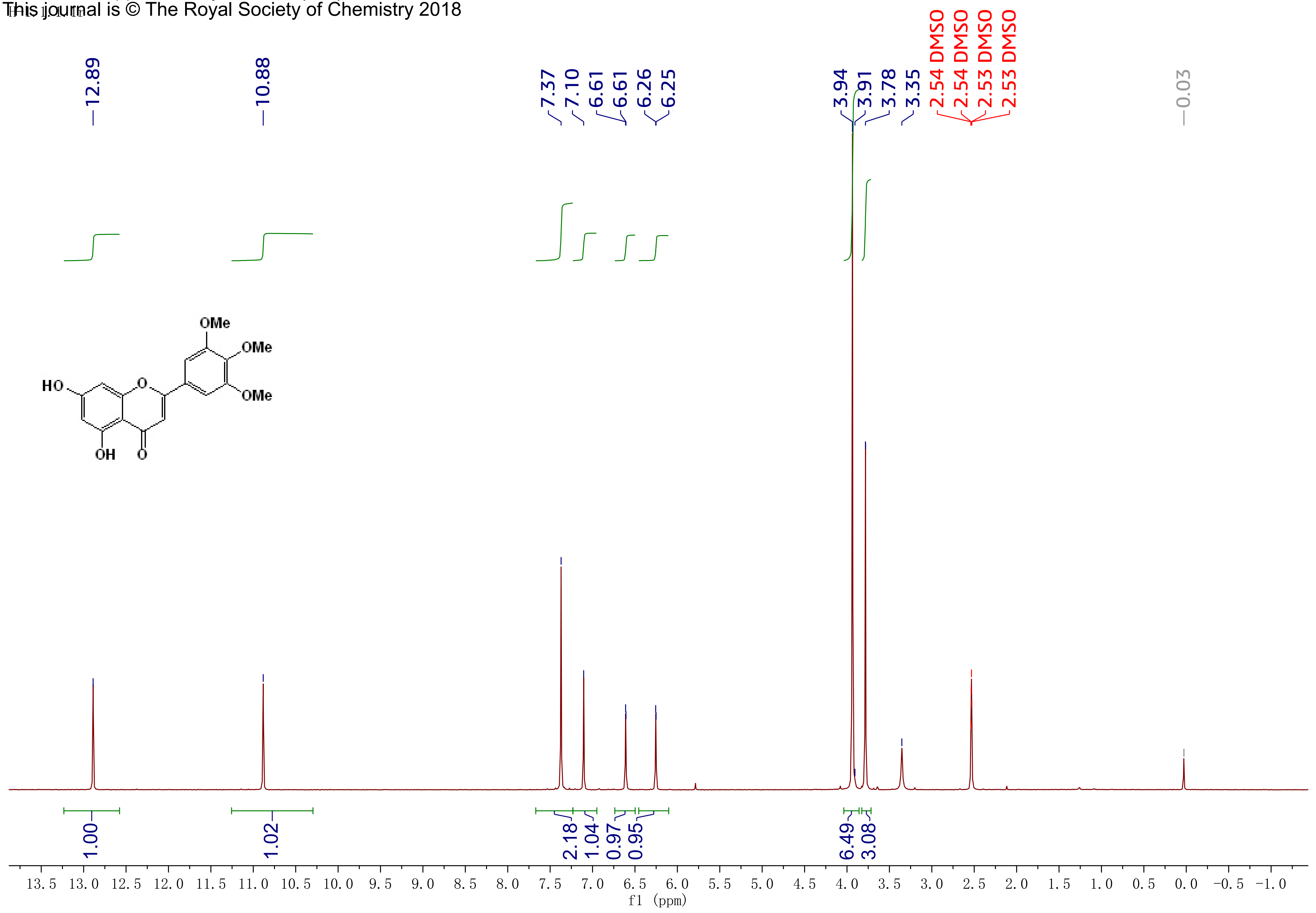

20171019 H-1.2.1.1r
Bruker AVANCEIII 400 20171019
C13 DMSO D:\\ DATA-2017 5

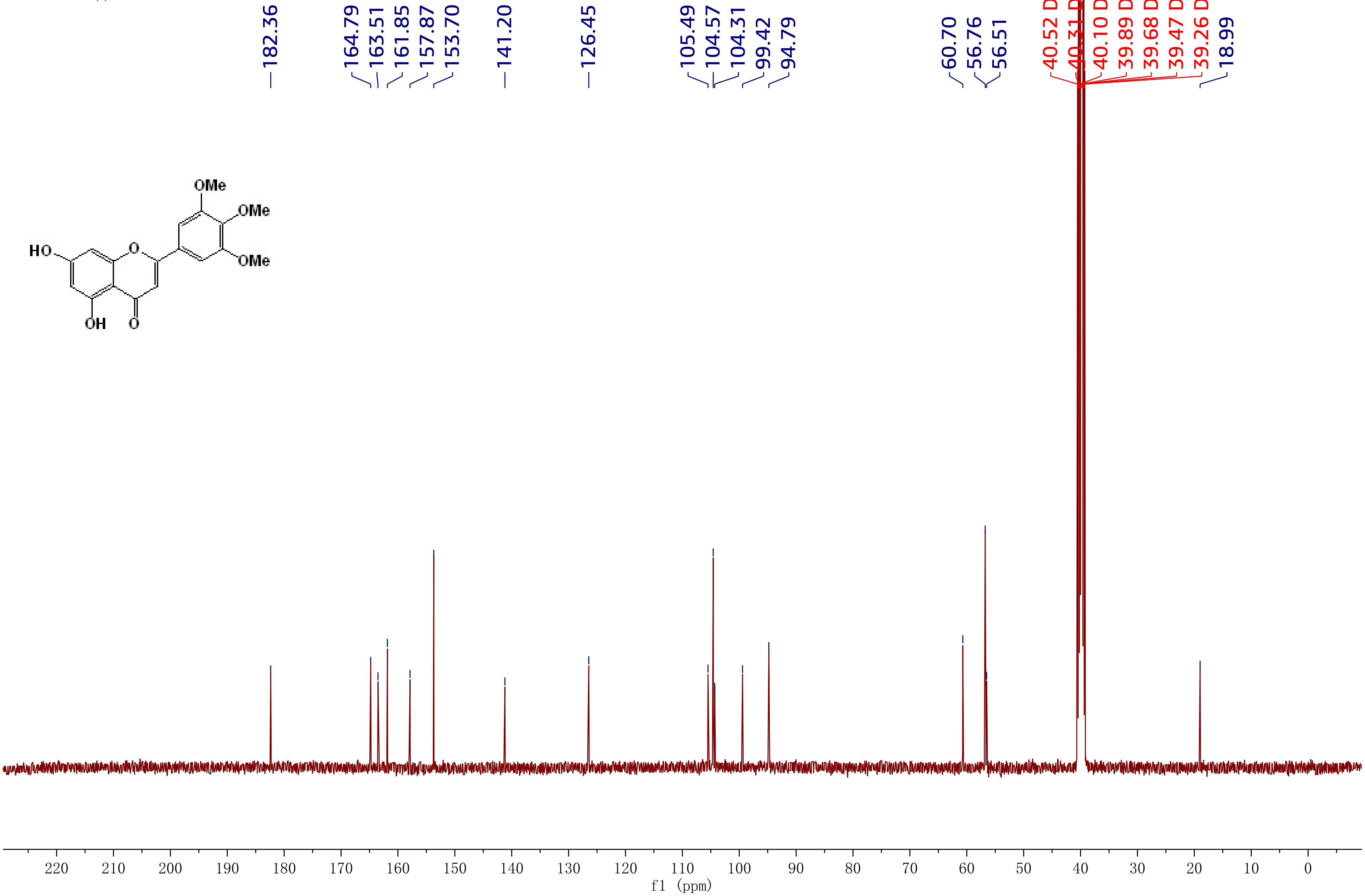

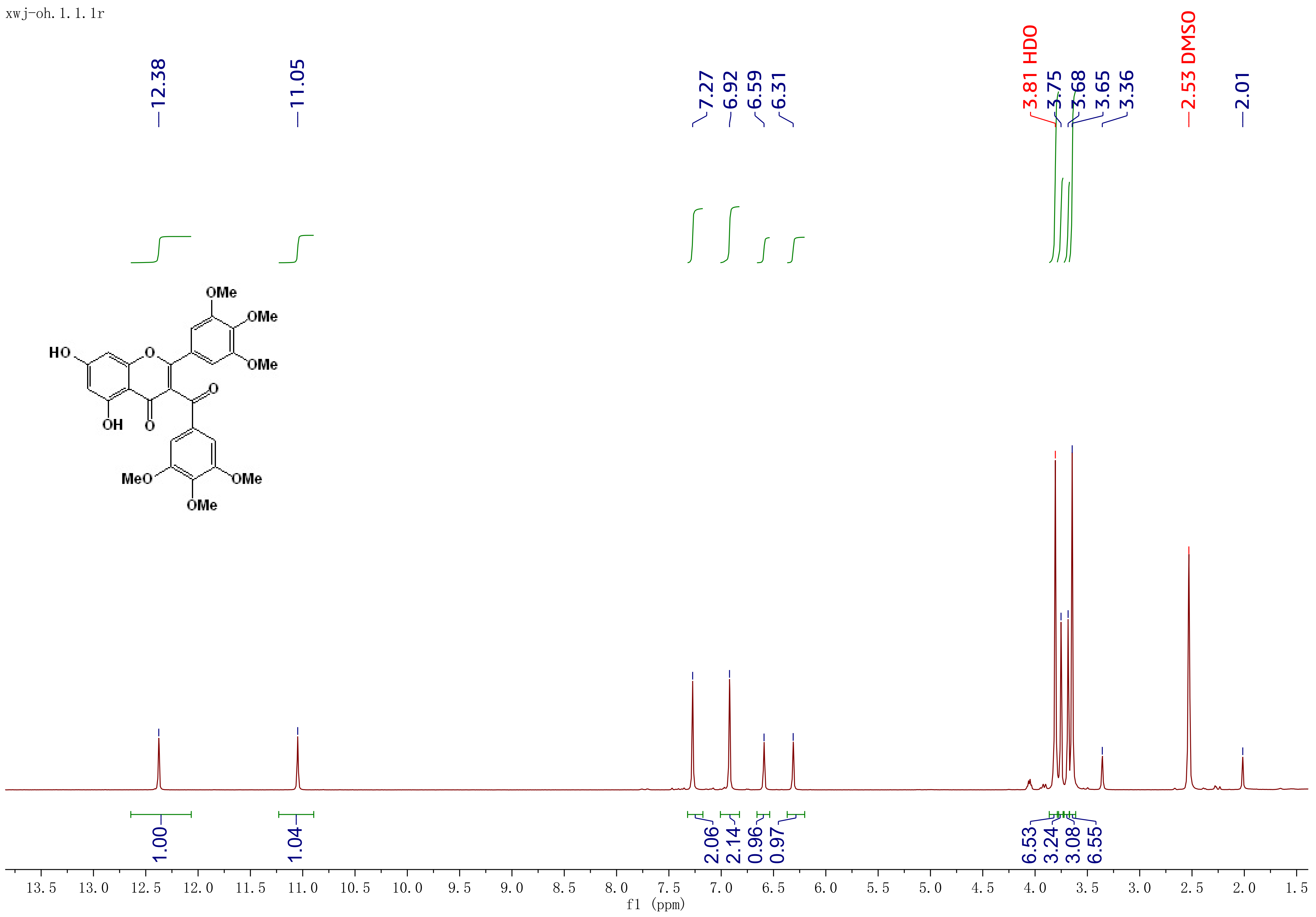

xwj-oh.1.1.1r
12.38
11.05
7.27
6.92
6.59
6.31
3.81 HDO
3.75
3.68
3.65
3.36
2.53 DMSO
2.01
OMe
OMe
HO
O
OMe
O
OH
O
MeO
OMe
OMe
1.00
1.04
2.06
2.14
0.96
0.97
6.53
3.24
3.08
6.55
13.5
13.0
12.5
12.0
11.5
11.0
10.5
10.0
9.5
9.0
8.5
8.0
7.5
7.0
6.5
6.0
5.5
5.0
4.5
4.0
3.5
3.0
2.5
2.0
1.5
f1 (ppm)

20161122-xwj-oh.1.1.1r
BRUKER AV-III-500 13C-NMR xwj-oh IN DMSO 2016.11.22
13C-NMR DMSO E:\\ wusong1 1

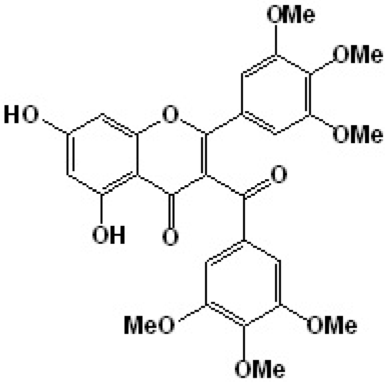

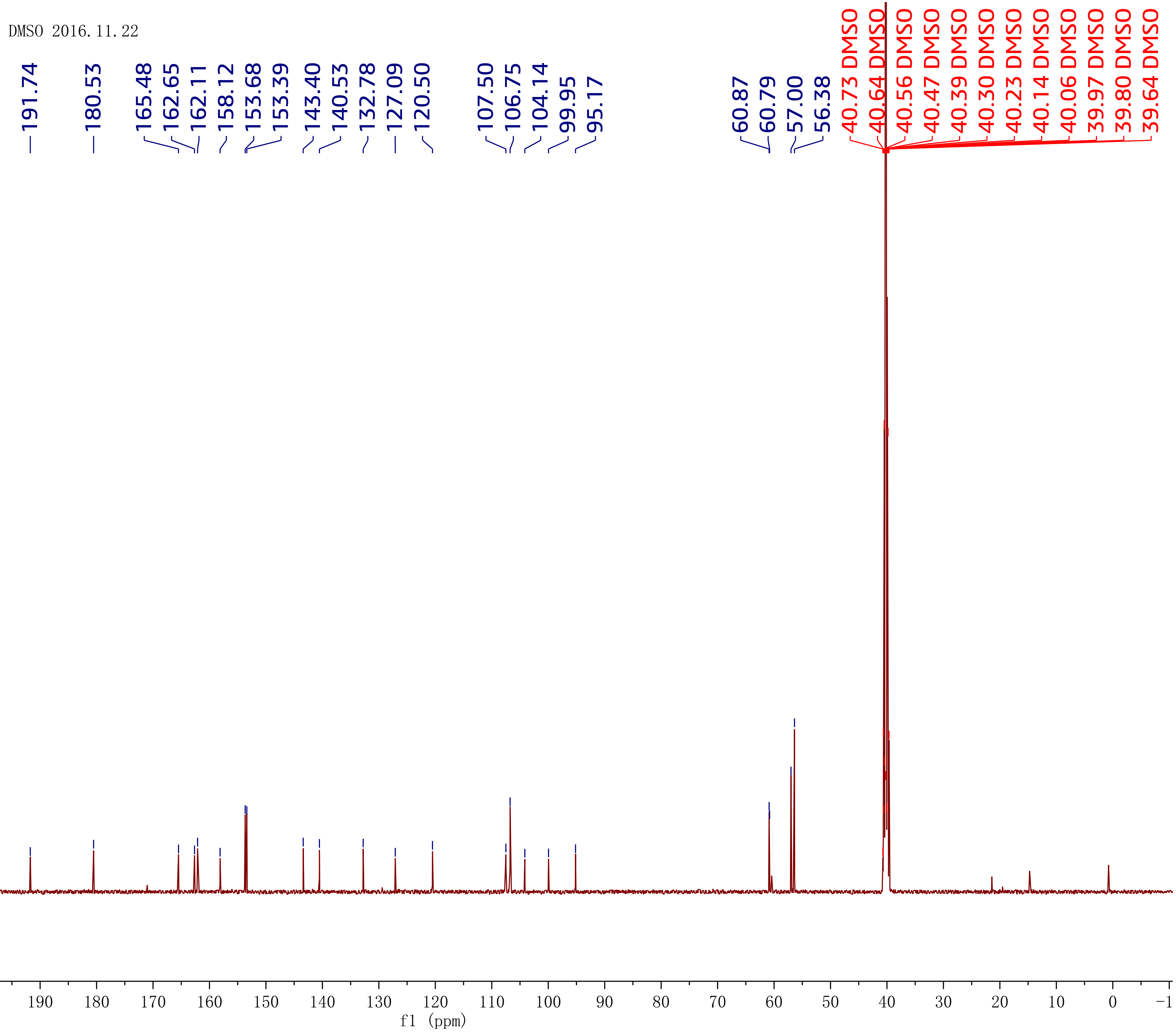

HEHE-4.1.1.1r

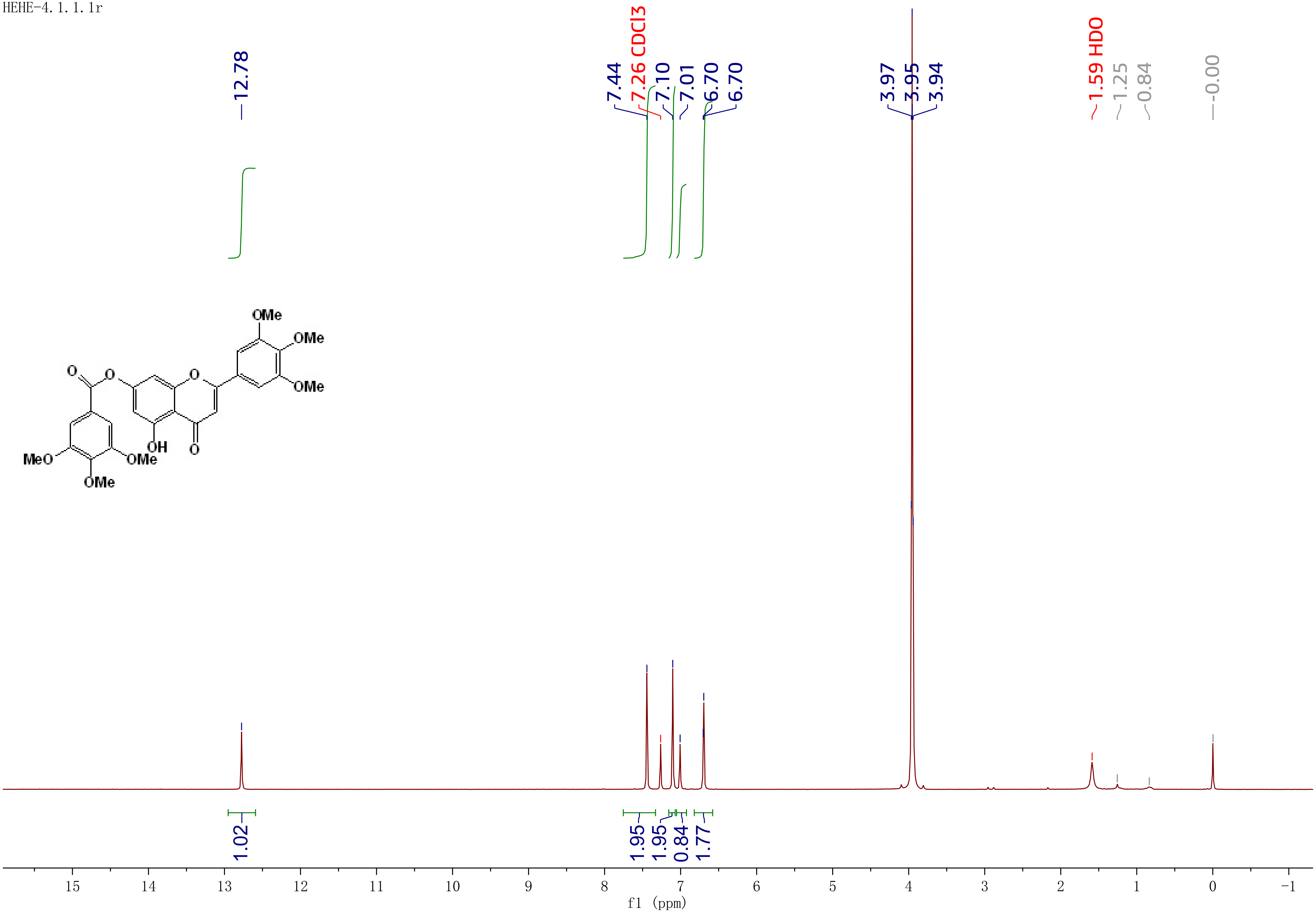

12.78
7.44
7.26 CDCl3
7.10
7.01
6.70
6.70
3.97
3.95
3.94
1.59 HDO
1.25
0.84
-0.00
1.02
1.95
1.95
0.84
1.77
OMe
OMe
OMe
O
O
O
MeO
OMe
OMe
OH
O
15 14 13 12 11 10 9 8 7 6 5 4 3 2 1 0 −1
f1 (ppm)

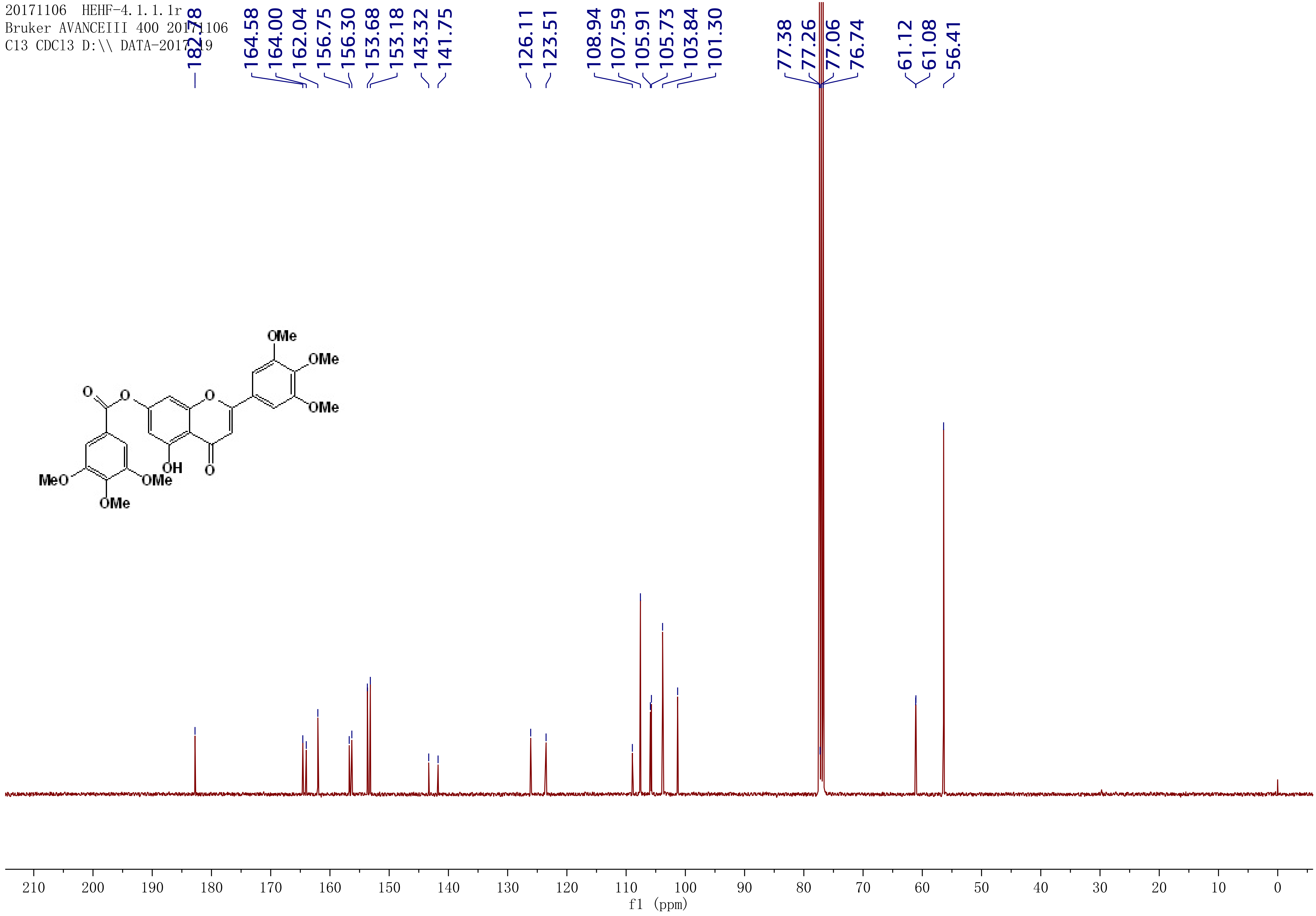

20171106 HEHF-4.1.1.1r
Bruker AVANCEIII 400 20171106
C13 CDCl3 D:\\ DATA-2017 19
182.78
164.58
164.00
162.04
156.75
156.30
153.68
153.18
143.32
141.75
126.11
123.51
108.94
107.59
105.91
105.73
103.84
101.30
77.38
77.26
77.06
76.74
61.12
61.08
56.41
OMe
OMe
OMe
O
O
O
MeO
OMe
OMe
OH
O
210 200 190 180 170 160 150 140 130 120 110 100 90 80 70 60 50 40 30 20 10 0
f1 (ppm)

HEHE-5.1.1.1r

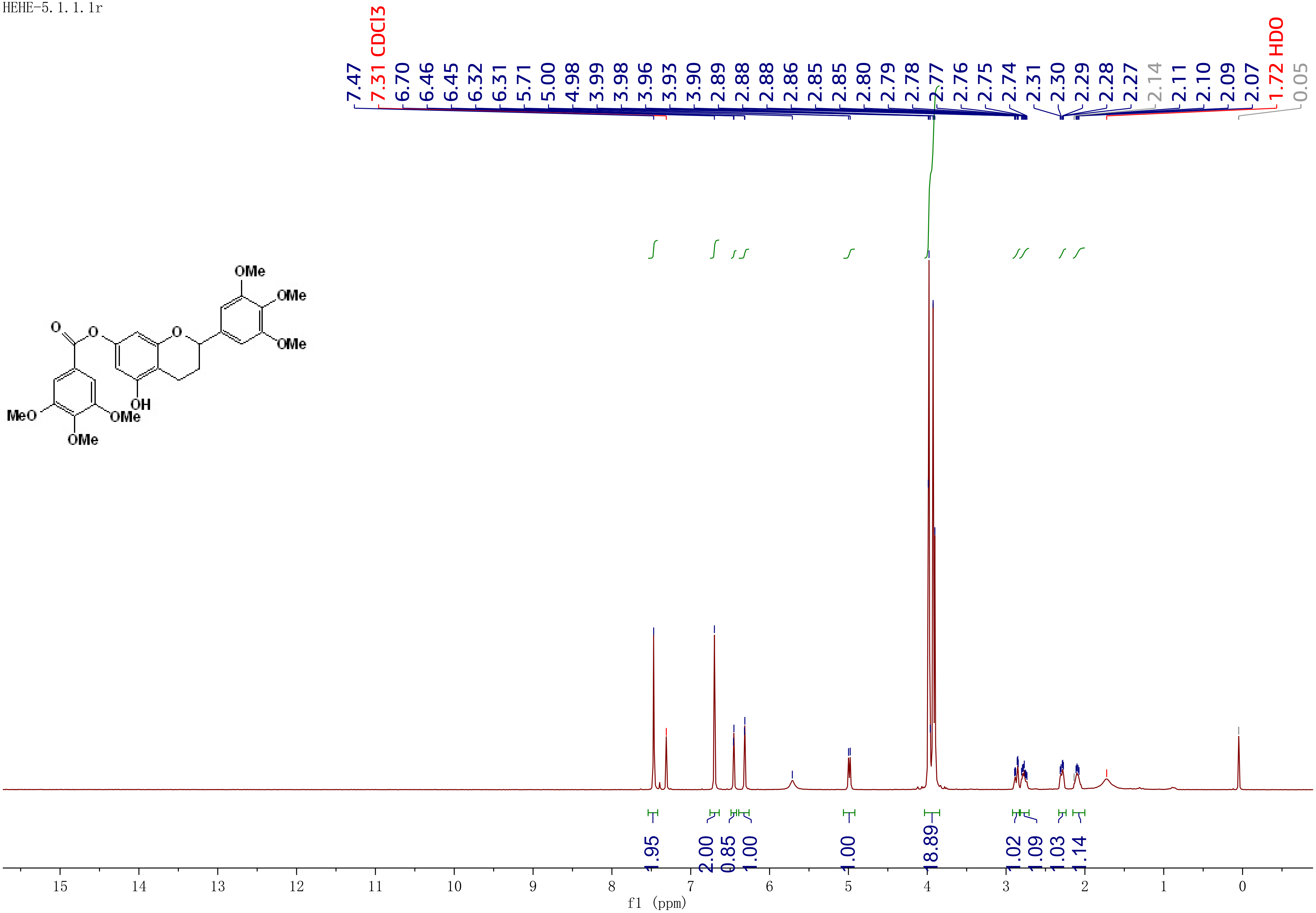
7.47
7.31 CDCl3
6.70
6.46
6.45
6.32
6.31
5.71
5.00
4.98
3.99
3.98
3.96
3.93
3.90
2.89
2.88
2.88
2.86
2.85
2.85
2.80
2.79
2.78
2.77
2.76
2.75
2.74
2.31
2.30
2.29
2.28
2.27
2.14
2.11
2.10
2.09
2.07
1.72 HDO
0.05
1.95
2.00
0.85
1.00
1.00
18.89
1.02
1.09
1.03
1.14
15
14
13
12
11
10
9
8
7
6
5
4
3
2
1
0
f1 (ppm)
OMe
OMe
O
O
O
OMe
MeO
OMe
OH
OMe

20171019 HEHF-5.2.1.1r
Bruker AVANCEIII 400 20171019
C13 CDCl3 D:\\ DATA-2017 8

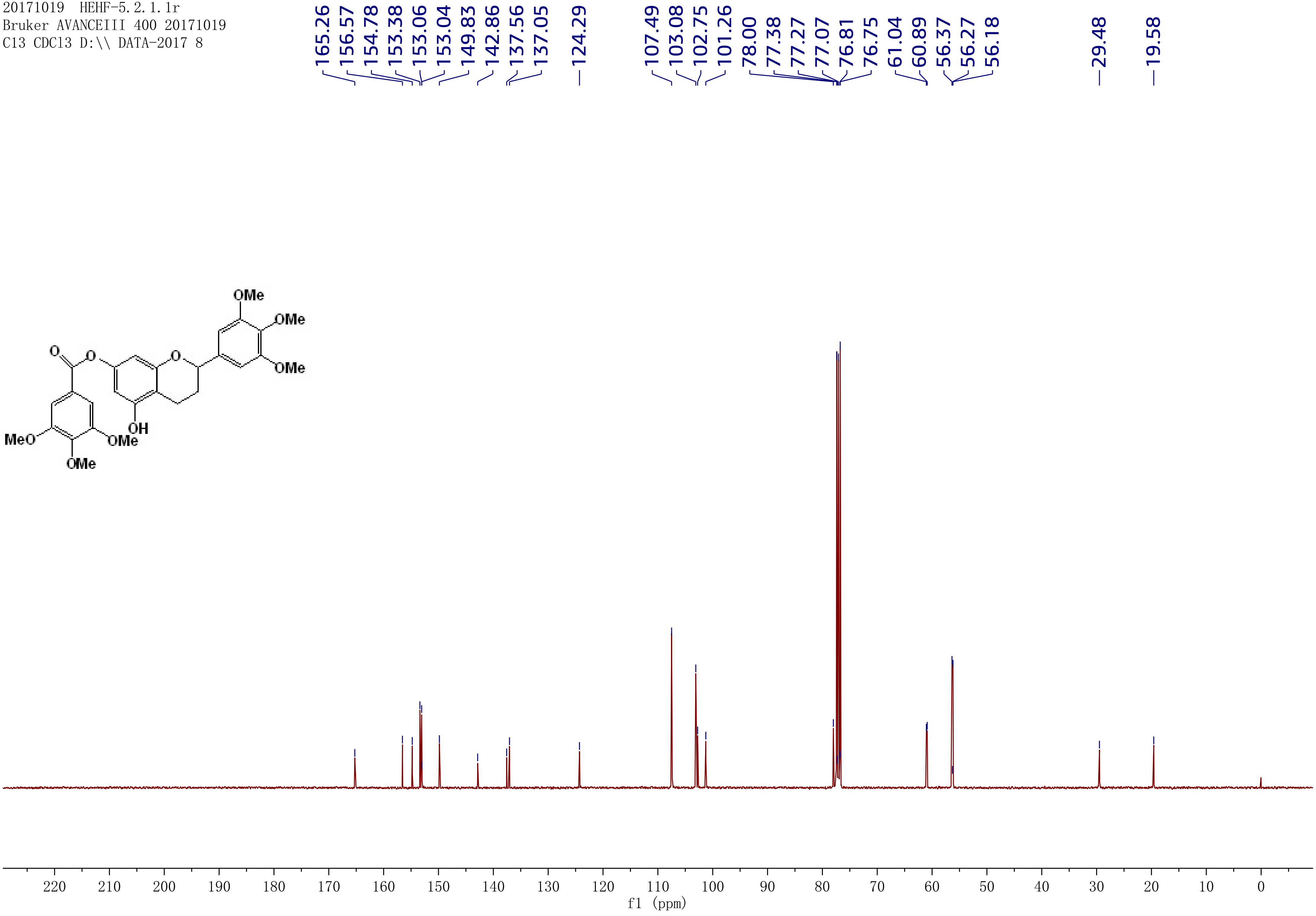

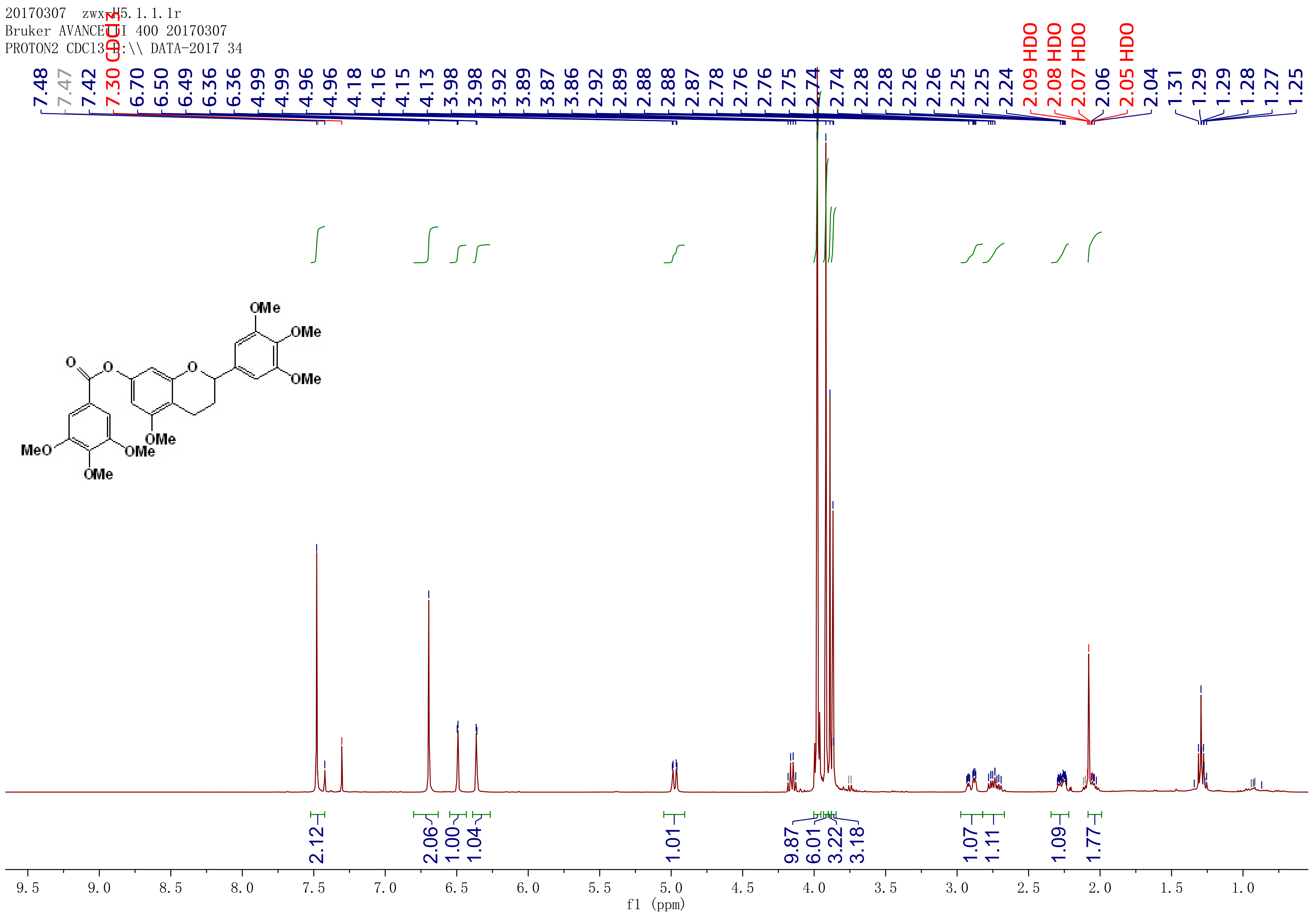
20170307 zwx-H5.1.1.1r
Bruker AVANCE III 400 20170307
PROTON2 CDCl3 D:\\ DATA-2017 34
CDCl3
HDO
OMe
O
MeO
f1 (ppm)

20170307 zwx-H5.2.1.1r
Bruker AVANCEIII 400 20170307
C13 CDCl3 D:\\ DATA-2017 34

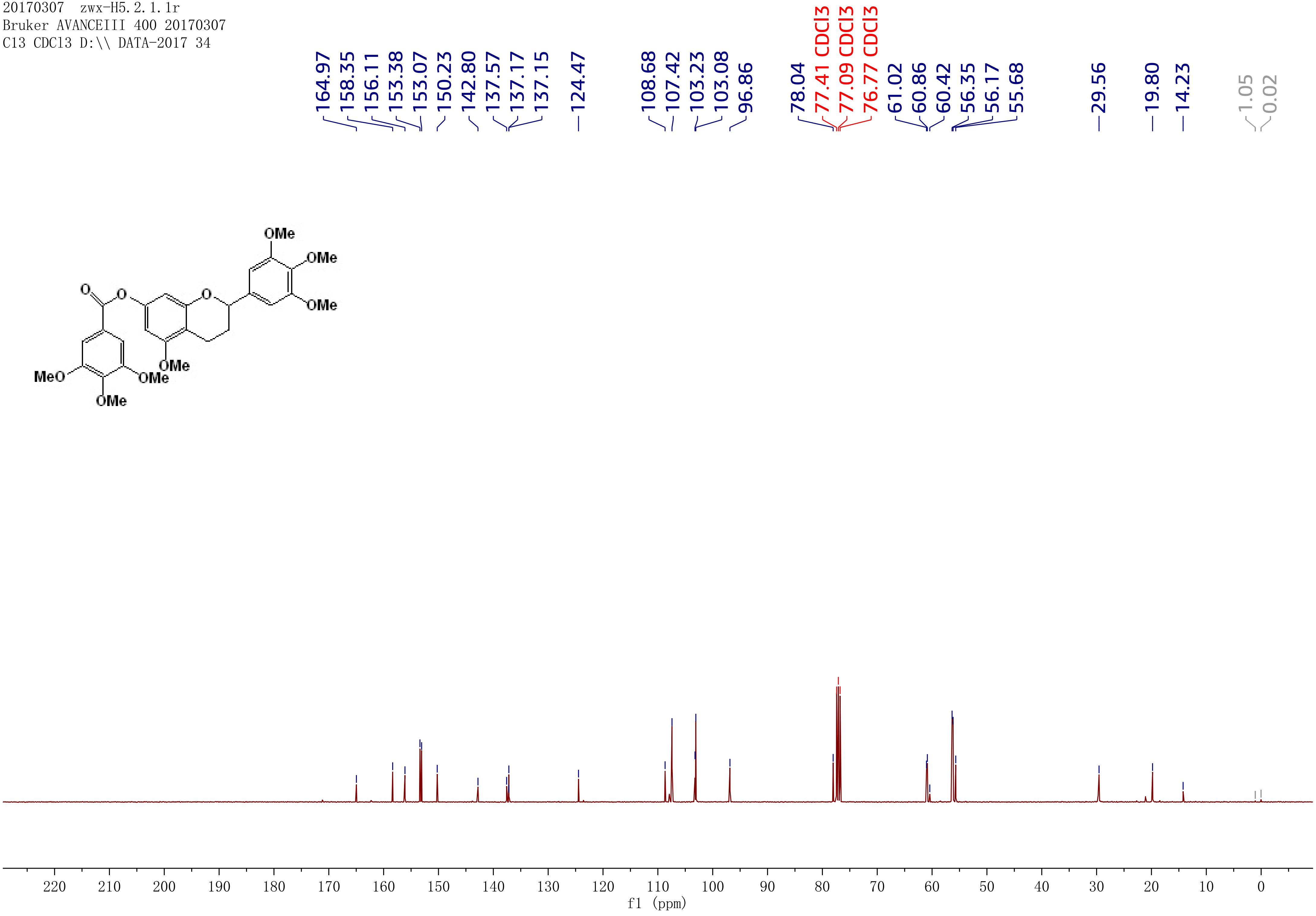

20171128 HEHE-1128.1.1.1r
Bruker AVANCEIII 400 20171128
PROTON2 MeOD D:\\ DATA-2017 19

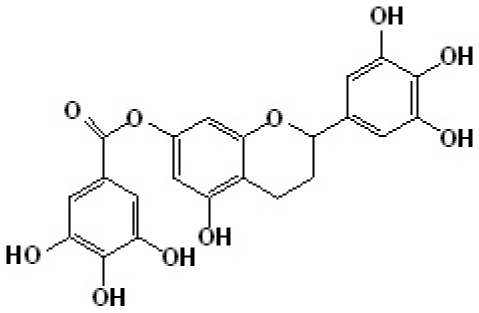

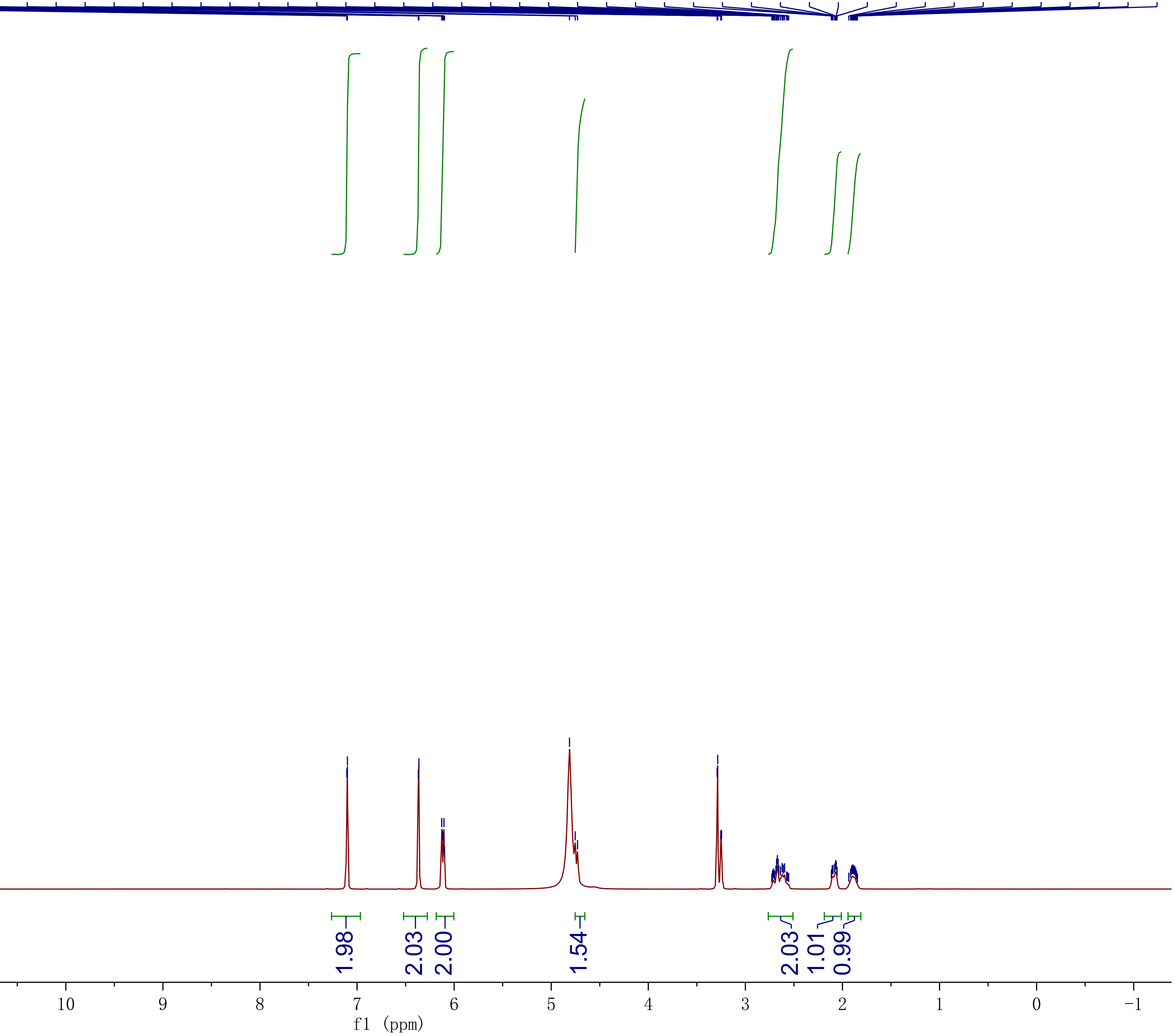

20171128 HEHF-1128.2.1.1r
Bruker AVANCEIII 400 20171128
C13 MeOD D:\\ DATA-2017 19

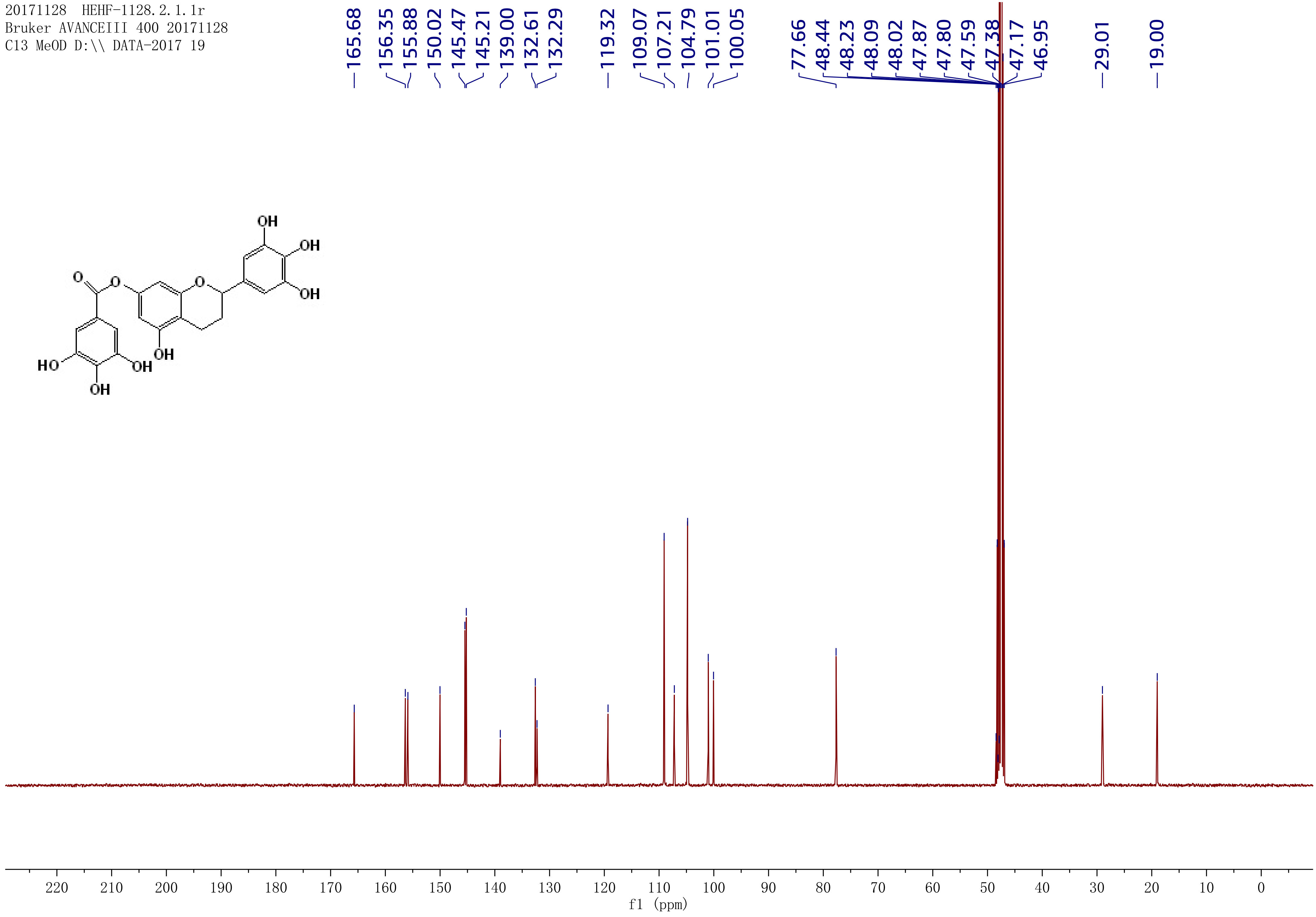

HEHF-Ac-20171117.1.1.1r

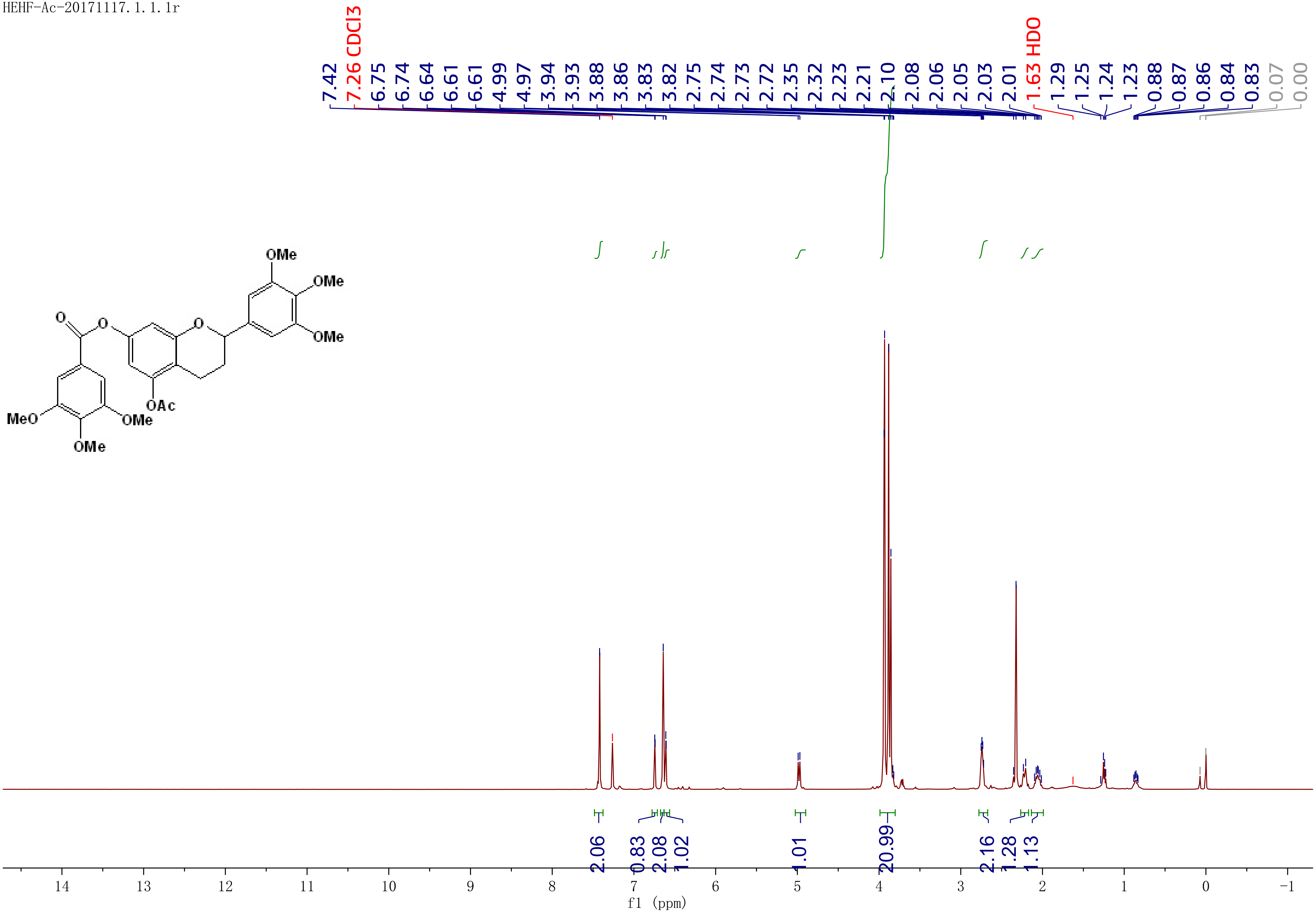

20171106 HEHF-Ac.1.1.1r
Bruker AVANCEIII 400 20171106
C13 CDCl3 D:\\ DATA-2017 18

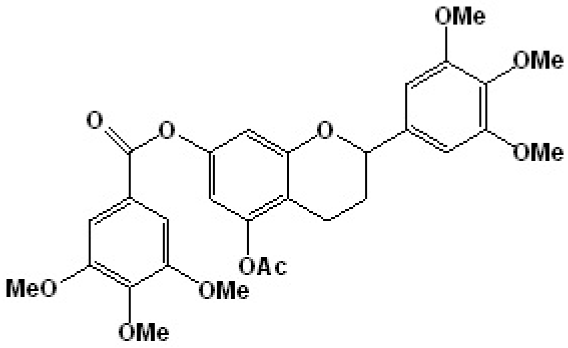

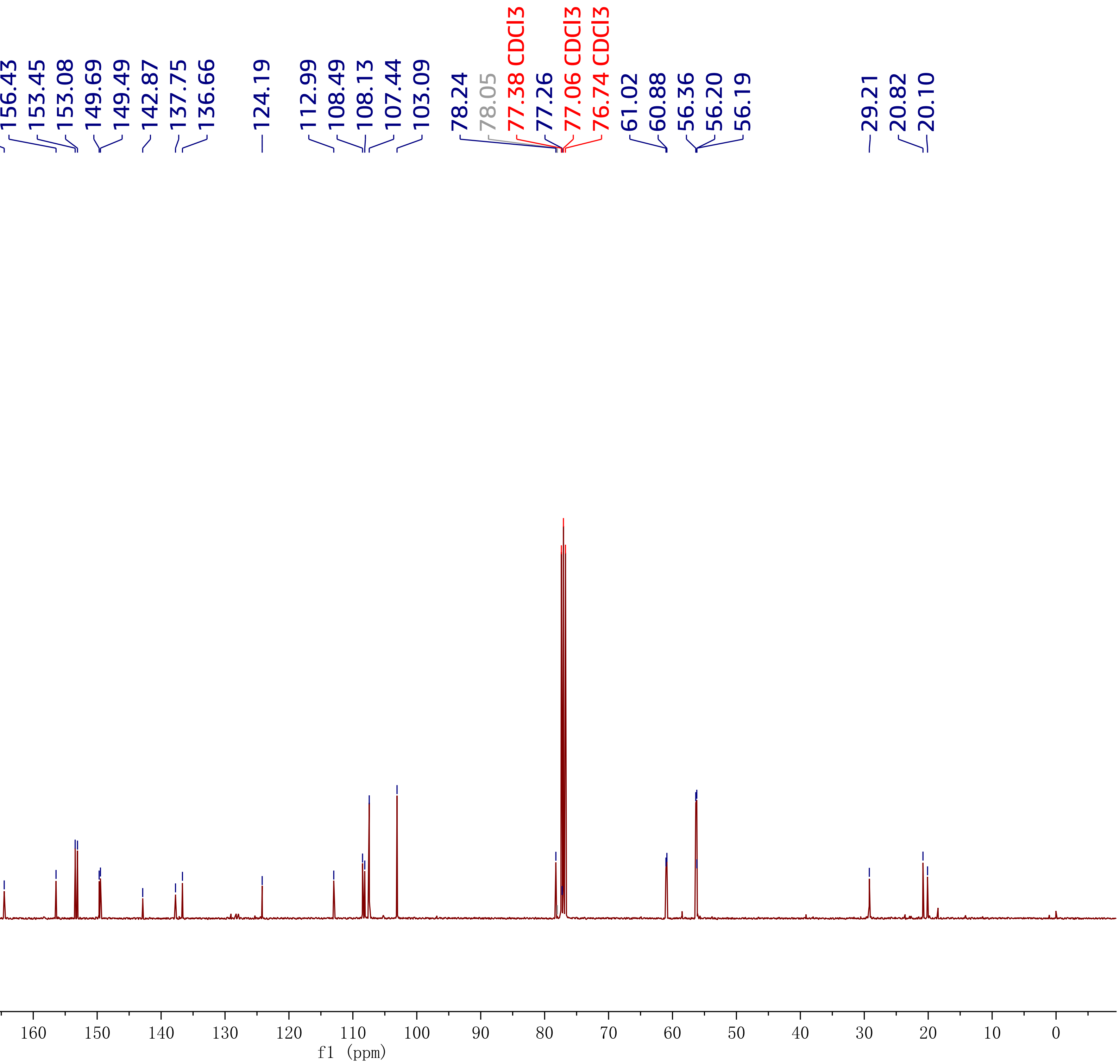

HEHF-byx-20171117.1.1.1r

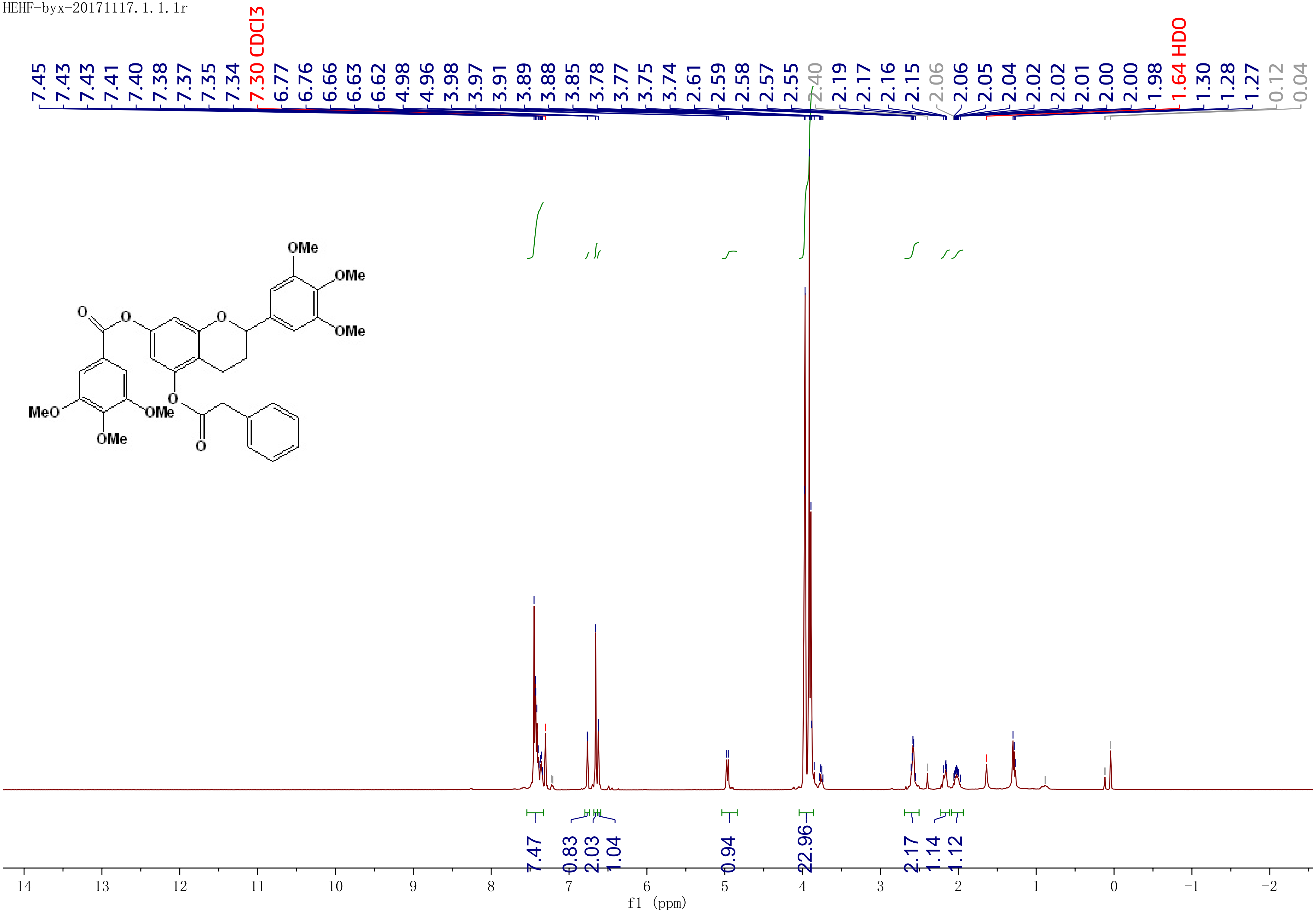

20171106 HEHF-BYX.1.1.1r
Bruker AVANCEIII 400 20171106
C13 CDCl3 D:\\ DATA-2017 20

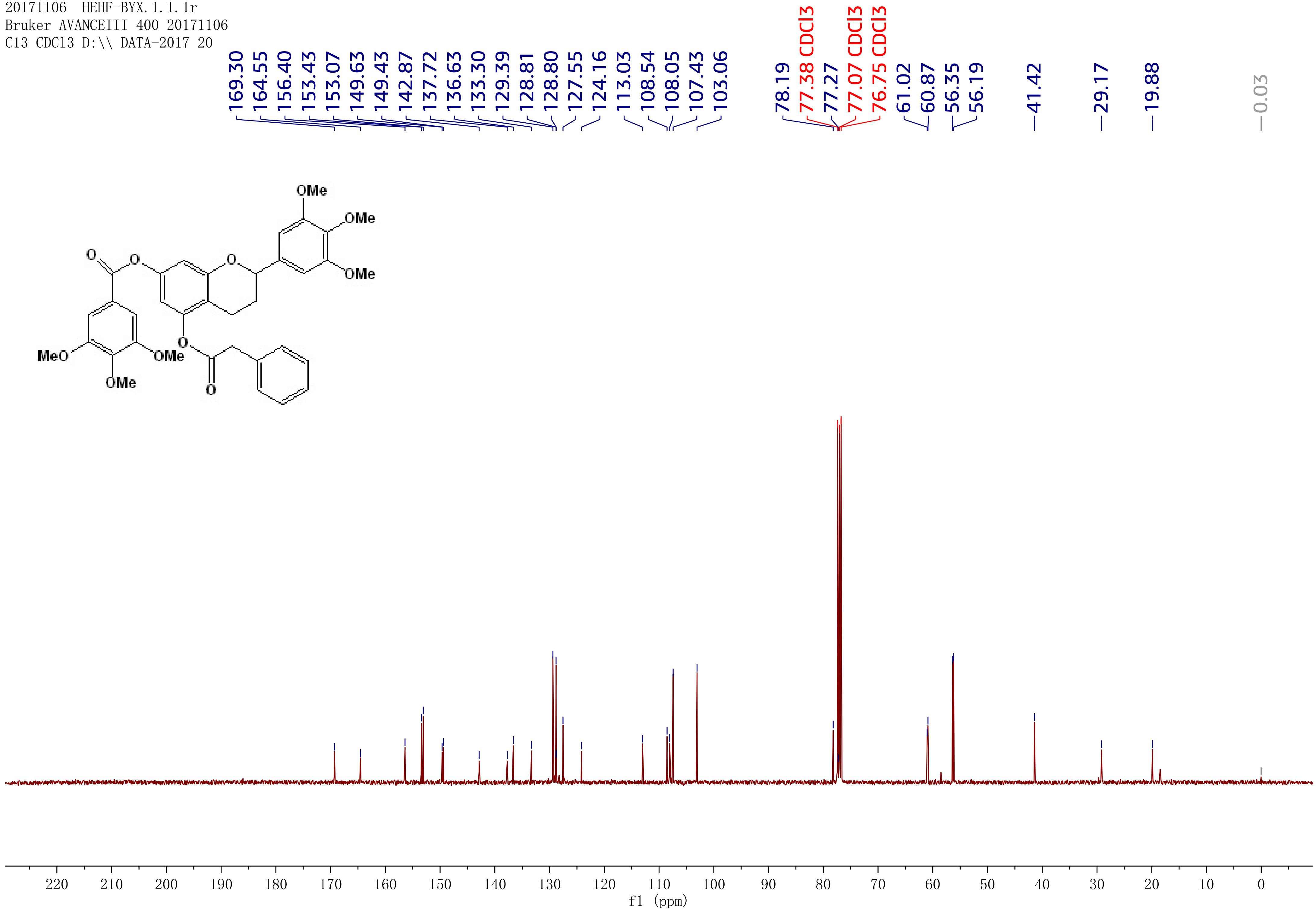

Supplement: RA-008-C8RA01606B-s001 [file RA-008-C8RA01606B-s001.pdf]
